# Measuring neck circumference to predict and detect haemorrhage following thyroid surgery. A case series and literature review

**DOI:** 10.1093/jscr/rjab089

**Published:** 2021-04-19

**Authors:** Mohsen A Ezzy, Moustafa H Elshafei, Mohamed A Sharaan

**Affiliations:** Department of General and Minimal Invasive Surgery, Nordwest Hospital, Frankfurt, Germany; Department of General and Minimal Invasive Surgery, Nordwest Hospital, Frankfurt, Germany; General and Minimal Invasive Surgery, Faculty of Medicine, Alexandria University, Egypt

## Abstract

Post-thyroidectomy bleeding is a fortunately rare but potentially life-threatening complication that may increase postoperative morbidity and hospital stay. In this case series, we demonstrate the relation between the measurement of neck circumference and haemorrhage following thyroid surgery and the value of this measurement in predicting post-thyroidectomy haemorrhage.

Currently, there is no simple and reliable method available for the early detection of post-thyroidectomy bleeding. Continuous pressure measurement could be a potential tool for the early detection of haemorrhage but is invasive, and more data are required to recommend threshold values for revision surgery. Early recognition and prompt surgical intervention are key to the management of cervical haematoma. Measurement of the neck circumference is a valuable adjunct tool in the early recognition of post-thyroidectomy bleeding. In this case series, we concluded that a 4-cm increase in neck circumference may trigger the clinical decision for cervical exploration.

## INTRODUCTION

Postoperative haemorrhage is a well-known complication of thyroid surgery and requires special attention since it may be life-threatening due to acute airway obstruction. The reported incidence of post-thyroidectomy haemorrhage is between 0.5 and 4.3% [1]. Approximately 40–60% of bleeding occurs within the first 8 h, and 80–85% occurs within the first 24 h following surgery [2].

The risk factors for post-thyroidectomy haemorrhage are old age, male sex, bilateral thyroidectomy, neck dissection, recurrent goiter, hypertension, Graves’ disease, coagulopathy, antithrombotic drug use, thyroid procedure in low-volume hospitals, gland size and the use of the clamp-and-tie technique rather than an energy device for haemostasis [1, 2].

The intraoperative application of the Valsalva manoeuvre is useful to detect minor bleeding in some patients during thyroidectomy but does not prevent postoperative haemorrhagic complications [3].

The routine use of drains does not prevent postoperative haematoma and is not a substitute for meticulous haemostasis. Moreover, several studies have suggested that the routine use of drains could increase the risk of infection, length of hospital stay, treatment costs and discomfort for the patient. Therefore, the current practice of post-surgical drainage for thyroidectomy does not offer any significant advantage and is considered controversial [4].

Only a few case reports have demonstrated the value of the neck circumference in detecting or following up on neck haematoma, e.g. blunt neck trauma and haematoma following carotid endarterectomy [5, 6].

In arthroscopic surgery, neck circumference measurements following shoulder arthroscopy are used as a diagnostic tool to indicate possible respiratory stress. In one study, the authors concluded that airway compromise is possible if the neck circumference increases by more than 4 cm [7].

Compartment pressure monitoring is a possible method for the early recognition of postoperative haemorrhage following thyroid surgery but is still invasive [8].

The key factors for the management of post-thyroidectomy haemorrhage include close observation, early detection and airway management. Surgical exploration and evacuation of the haematoma is indicated for patients with symptoms such as respiratory distress, neck pain or pressure and dysphagia [9].

Our aim was to evaluate the relationship between postoperative cervical haematoma and neck circumference.

## CASE SERIES

### Case 1

A 66-year-old male patient was admitted to our surgical department for elective total thyroidectomy with a symptomatic non-toxic multinodular goiter. He was recently diagnosed with atrial fibrillation and was taking rivaroxaban, which was suspended 48 hours before surgery and bridged to heparin subcutaneous therapy. He had known hypertension, coronary artery disease and type 2 diabetes mellitus. Physical examination revealed palpable neck swelling, which was movable with swallowing. The neck ultrasound showed an enlarged thyroid gland with multiple nodules on both sides; the thyroid volume was 26 mL. The preoperative thyroid function test, parathyroid hormone and serum calcitonin were within normal ranges.

The patient underwent total thyroidectomy. The parathyroid glands and recurrent nerves were recognized and preserved. After meticulous haemostasis and intraoperative Valsalva manoeuvre, the wound was closed. No drain was placed. The patient was sent to the recovery room and then to the normal ward.

The neck circumference was regularly measured at different intervals as follows: the preoperative measurement was 42 cm; immediately postoperative, 42.5 cm; 30 min, 43 cm; 60 min, 43 cm; 90 min, 44 cm; 120 min, 44 cm; 4 h, 45 cm; and 6 h, 47 cm. At this point, the patient complained of a pressure sensation in his neck but no respiratory distress or hypoxia.

The patient was transferred immediately to the operating room (OR). Our anaesthesiologist experienced no difficulties in intubation. After surgical draping, the wound was opened, and cervical exploration was carried out, which revealed a haematoma deep to the strap muscles that appeared as a diffuse oozing haemorrhage. Following the removal of several clots, irrigation of the surgical bed, meticulous haemostasis and application of thrombin-coated collagen (TachoSil) covering the entire bleeding surface were performed. Two drains were left in the thyroid bed. The patient was extubated and sent to the recovery room and later to the surgical ward. The postoperative course was uneventful. Anticoagulant was restarted 12 h after surgery (heparin twice daily). Oral intake was gradually increased as tolerated. The drains were removed on postoperative Day 2. Histopathology returned benign. The patient was discharged on postoperative day 4 in good general condition.

### Case 2

A 71-year-old female patient, known to have arterial hypertension and coronary artery disease and on aspirin therapy, was admitted to the hospital for elective total thyroidectomy with a symptomatic non-toxic multinodular euthyroid goiter.

Physical examination revealed palpable neck swelling. The neck ultrasound showed an enlarged thyroid gland with multiple nodules on both sides; the thyroid volume was 22 ml. The preoperative thyroid function test, parathyroid hormone and serum calcitonin were within normal ranges.

The patient underwent a total thyroidectomy. The parathyroid glands and recurrent nerves were recognized and preserved. After meticulous haemostasis and an intraoperative Valsalva manoeuvre, the wound was closed. No drain was placed. The patient was sent to the recovery room and then to the normal ward.

The neck circumference was regularly measured at different intervals as follows: the preoperative measurement was 37 cm; immediately postoperative, 38 cm; 30 min, 38 cm; 60 min, 38 cm; 90 min, 39 cm; 120 min, 39 cm, 4 h, 39 cm; 6 h, 40 cm; and 8 h, 41 cm. At this point, the patient experienced a pressure sensation and tightness in her neck but no respiratory distress or hypoxia.

The patient was transferred immediately to the OR. No difficulties in intubation were reported by our anaesthesiologist. After surgical draping, the wound was opened and cervical exploration was carried out, which revealed a haematoma deep to the strap muscles. Blood clots were removed, the surgical bed was irrigated and bleeding was identified from the small venous branch of the middle thyroid vein. After ligation of the bleeder and meticulous haemostasis, drains were placed in the thyroid bed. The patient was extubated and shifted to the recovery room and later to the surgical ward. The postoperative course was uneventful. Oral intake was gradually increased as tolerated. The drains were removed on postoperative Day 2. Histopathology returned benign. The patient was discharged on postoperative Day 4 in good general condition.

### Case 3

A 62-year-old female patient, known to have type 2 diabetes mellitus and a post-transient ischaemic attack and who was on aspirin therapy, was admitted to the hospital for elective right hemithyroidectomy with a 1,8 cm thyroid nodule.

Physical examination was unremarkable. The neck ultrasound showed a solitary, rounded encapsulated lesion measuring 1.8 cm in the right lobe, and the thyroid volume was 19 ml. The preoperative thyroid function test, parathyroid hormone and serum calcitonin were within normal ranges. The fine needle aspiration cytology revealed Bethesda category three nodules.

The patient underwent right hemithyroidectomy. The parathyroid glands and recurrent nerves were recognized and preserved. After meticulous haemostasis and an intraoperative Valsalva manoeuvre, the wound was closed. No drain was placed. The patient shifted to the recovery room and was later moved to the normal ward.

The neck circumference was regularly measured at different intervals as follows: the preoperative measurement was 35 cm; the immediately postoperative measurement, 35.5 cm; 30 min, 35.5 cm; 60 min, 36 cm; 90 min, 36 cm; 120 min, 37 cm; 4 h, 37 cm; and 6 h, 38 cm. At this point, the patient experienced a pressure sensation and skin discolouration in her neck, whose circumference measured 40 cm, but no respiratory distress or hypoxia.

The patient was transferred immediately to the OR. The intubation was smooth and without difficulty. After surgical draping, the wound was opened, and cervical exploration was carried out, which revealed a haematoma superficial to the strap muscles. Blood clots were removed, and a deeper exploration was performed by opening the cervical linea alba. No deep haematoma was found. After irrigation, meticulous haemostasis and application of CFTP (TachoSil), drains were placed in the thyroid bed. The patient was extubated and shifted to the recovery room and later to the surgical ward. The postoperative course was uneventful. Oral intake was gradually increased as tolerated. The drains were removed on postoperative Day 2. Histopathology returned thyroid adenoma. The patient was discharged on postoperative Day 4 in good general condition.

## DISCUSSION

Currently, there is no simple and reliable method available for the early detection of postoperative bleeding. Continuous pressure measurement could be a potential tool for the early detection of postoperative bleeding in thyroid surgery but is invasive, and more data are required to recommend threshold values for revision surgery [8].

The neck circumference is easily measured non-invasively and inexpensively, but to date, no study has revealed the correlation between neck circumference and post-thyroidectomy haemorrhage.

To our knowledge, this is the first case series evaluating the relation between neck circumference and postoperative cervical haematoma following thyroid surgery.

**Figure 1:**
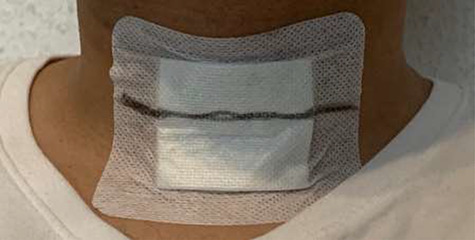
Measuring neck circumference after thyroid surgery.

The neck circumference was measured for all patients using non-stretchable plastic tape, with increments down to 0.1 cm. The patients were asked to sit erect or in Fowler’s position with the head positioned at the Frankfort horizontal plane. The superior border of the tape measure was placed just below the laryngeal prominence and applied perpendicular to the long axis of the neck (Fig. 1).

The measurements were carried out by one person preoperatively, immediately postoperatively, and 30 min, 60 min, 90 min, 120 min, 4 h, 6 h, 8 h, 24 h and 48 h after the operation.

There was a 0.5–1 cm difference between preoperative and immediate postoperative neck circumference due to wound dressing. Therefore, it is important to make comparisons with the immediate postoperative value rather than the preoperative value.

Patients who experienced postoperative bleeding had an increase in the postoperative neck circumference of 4 cm or more and subsequently became symptomatic.

In our series, bleeding occurred within 8 h postoperatively, and all patients were under antithrombotic agents.

In our series, no patient developed respiratory distress, died or required bedside haematoma evacuation, and bleeding was associated with a minimal drop in haemoglobin (1–1,5 g/dl). No blood transfusion was required.

It is judicious to actively manage the patient before airway compromise occurs by early evacuation of the haematoma either at bedside or in the operating theatre.

All patients were revised with meticulous haemostasis and drain placement. The estimated blood loss in the revision surgery was between 40 and 100 ml.

The topical haemostatic agent collagen fibrinogen and thrombin patch (CFTP) had previously been used in thyroid surgery and has shown efficacy in controlling oozing bleeding as well as in reducing the drainage volume [10].

All patients recovered well and were discharged 4 days after the revision surgery (mean length of hospital stay following thyroid surgery at our institution was 2 days).

Measurement of the neck circumference is a convenient method that is easy to perform, non-invasive, inexpensive and widely available.

In conclusion, early recognition and prompt surgical intervention are key to the management of cervical haematoma. Measurement of the neck circumference is a valuable adjunct tool in the early recognition of postoperative thyroid bleeding. In this case series, we concluded that a 4 cm increase in neck circumference may trigger the clinical decision for cervical exploration.

## CONFLICT OF INTEREST STATEMENT

None declared.

## FUNDING

This paper did not receive any specific grant from funding agencies in the public, commercial, or not-for-profit sectors.

## ETHICS APPROVAL

Not applicable. This is a case series based on the clinical notes of an individual patient, and written consent for publication has been obtained from the patient.

## CONSENT

Written informed consent was obtained from the patient for the publication of this case series and accompanying image.

## REGISTRATION OF RESEARCH STUDIES

Not Applicable.

## GUARANTOR

Dr. Mohsen Ezzy.
